# CT-perfusion measurements in pancreatic carcinoma with different kinetic models: Is there a chance for tumour grading based on functional parameters?

**DOI:** 10.1186/s40644-016-0100-6

**Published:** 2016-12-15

**Authors:** Sven Schneeweiß, Marius Horger, Anja Grözinger, Konstantin Nikolaou, Dominik Ketelsen, Roland Syha, Gerd Grözinger

**Affiliations:** Department of Diagnostic Radiology, Eberhard-Karls-University, Hoppe-Seyler-Str.3, 72076 Tübingen, Germany

**Keywords:** Pancreatic carcinoma, Volume perfusion-CT, Kinetic calculation models, Grading

## Abstract

**Background:**

To evaluate the interchangeability of perfusion parameters obtained with help of models used for post-processing of perfusion-CT images in pancreatic adenocarcinoma and to determine the mean values and ranges of perfusion in different tumour gradings.

**Methods:**

Perfusion-CT imaging was performed prospectively in 48 consecutive patients with pancreatic adenocarcinoma. In 42 patients biopsy-proven tumor grading was available (4 × G1/24 × G2/14 × G3/6× unknown). Images were post-processed using a model based on the maximum-slope (MS) approach (blood flow-BFMS) + Patlak analysis (P) (blood volume [BVP] and permeability [k-transP]), as well as a model with deconvolution-based (D) analysis (BFD, BVD and k-transD). 50 mL contrast agent were applied with a delay time of 7 s. Perfusion parameters were compared using intraclass correlation coefficient (ICC), the Wilcoxon matched-pairs test and Bland-Altman plots.

**Results:**

Forty eight VOIs of tumours were outlined and analysed. Moderate to good ICC values were found for the perfusion parameters (ICC = 0.62–0.75). Wilcoxon matched-pairs revealed significantly lower values (*P* < .001 and 0.008), for the BF and BV values obtained using the maximum-slope approach + Patlak analysis compared to deconvolution based analysis. For k-trans measurement, deconvolution revealed significantly lower values (*P* < 0.001). Different histologic subgroups (G1-G3) did not show significantly different functional parameters.

**Conclusion:**

There were significant differences in the perfusion parameters obtained using the different calculation methods, and therefore these parameters are not directly interchangeable. However, the magnitude of pairs of parametric values is in constant relation to each other enabling the use of any of these methods. VPCT parameters did not allow for histologic classification.

## Background

Pancreatic cancer is one of the leading malignancies of the digestive tract [1]. It also has the poorest prognosis of all gastro-intestinal cancers. Over the last decades, there has been a continuous increase in diagnosed patients in Western industrialized nations. Only 2% of annually diagnosed patients outlive the following 5 years [2]. The most common pancreatic cancer is the adenocarcinoma of the pancreatic head [1]. Tumour characterization and accurate delineation is essential for staging and pre-operative planning, as in addition to TNM staging, the degree of malignancy, called tumour grading is a decisive parameter of survival [3, 4]. So far, tumour grading is done histologically, which requires invasive biopsy sampling. Hence the development of a non-invasive method of tumour grading would be highly desirable.

Volume perfusion CT (VPCT) is a relatively new modality that has been increasingly used for oncologic imaging over the last years [5]. Based on repetitive scanning of a tissue volume after contrast injection, VPCT enables the measurement of functional parameters of tumour vascularity like blood flow (BF), blood volume (BV) and the permeability of capillaries (permeability surface area product, or k-trans).

Recent studies show the capability of perfusion CT to evaluate tumour vascularization and monitor chemotherapy, radiation therapy or even effect of novel functional drugs affecting tumour environment and angiogenesis [6–10]. One preliminary study suggested that even a non-invasive tumour grading with VPCT might be possible in the case of pancreatic adenocarcinoma [6].

There are different calculation methods for the post-processing of perfusion-CT images: the compartment analysis assuming one (maximum slope) or two compartments (Patlak) and the deconvolution analysis. In other tumour tissues, several studies have already demonstrated significant differences in the calculated perfusion values between the different mathematical models [11–13].

For this purpose, reliability of CT-perfusion data is imperative. Besides an optimized CT-examinational protocol, standardization of perfusion quantification methods (post-processing) is essential, as well as knowledge about comparability of results using these different mathematical methods for perfusion calculation.

However, for reproducibility of studies and for determining cut-off values it is important to know if the different models deliver comparable results. For this reason this study explored for pancreas adenocarcinoma to which extent both models (maximum slope + Patlak and deconvolution) are comparable and if these functional parameters allow for a reliable tumour grading.

## Methods

### Clinical data

Inclusion criteria for the VPCT study were: Patients with suspicion of pancreatic cancer before treatment who agreed to take part in this study after informed consent. Exclusion criteria for VPCT were: Poor kidney function (GFR < 45 ml/min), pregnancy, allergy to contrast agent or iodine and incompliant patient unable to hold their breath.

Between September 2011 and November 2014, a total of 48 patients (28 male, 20 female; mean age: 69 ± 9 years, range: 39–84, respectively) were eligible for VPCT data analysis and prospectively enrolled in the study. In 42 of these patients, histologic data was available. In 4 cases patients refused a biopsy. In two cases, the biopsy did not contain sufficient diagnostic specimen for the pathologist. However, in time diagnosis of pancreatic cancer could be clearly made due to the presence of tumour markers and available imaging.

VPCT of the entire pancreas was performed. All biopsy specimens were examined by a pathologist and graded according to the AJCC Classification [14] and as described by Hruban et al.[15].

### CT Perfusion scanning technique

All examinations were performed on a 128-row CT scanner (Somatom Definition AS+, Siemens Healthcare, Forchheim, *Germany*). The CT protocol consisted of a non-enhanced abdominal low-dose CT (NECT) (40 mAs, 100 kV, 5.0 mm slice thickness, collimation 128*0.6 mm, tube rotation time 0.5 s, pitch 0.6), which was obtained to localize the pancreas. Subsequently, a VPCT of the tumour using adaptive spiral scanning technique was performed. In the adaptive spiral mode the z-range is scanned continuously with a shuttle movement of the patient table. Perfusion parameters were: 80 kV, 100/120mAs (for patients </> 70 kg, respectively), collimation 64 × 0.6 mm with z-flying focal spot (Z coverage 6.9 cm) and 26 CT-whole coverages of the pancreas within a total scan time of 40s. Patients were asked to resume shallow breathing for the entire duration of the study. 50 ml Ultravist 370 (Bayer Vital Leverkusen, Germany) were injected at a flow rate of 5 mL/s in an antecubital vein followed by a saline flush of 50 ml NaCl at 5 mL/s, and a fixed start delay of 7 s. Contrast medium was administered by using a dual-head pump injector (Stellant, Medtron, Saarbruecken, Germany). One set of axial images with a slice thickness of 3 mm for perfusion analysis was reconstructed without overlap, using a smooth tissue convolution kernel (B10f). All images were transferred to an external workstation (Multi-Modality Workplace, Siemens) for analysis. The mean effective whole-body dose values for VPCT examinations of the pancreas are estimated 7.0 mSv for men and 7.1 for women [16].

### CT Perfusion analysis

**Fig. 1:**
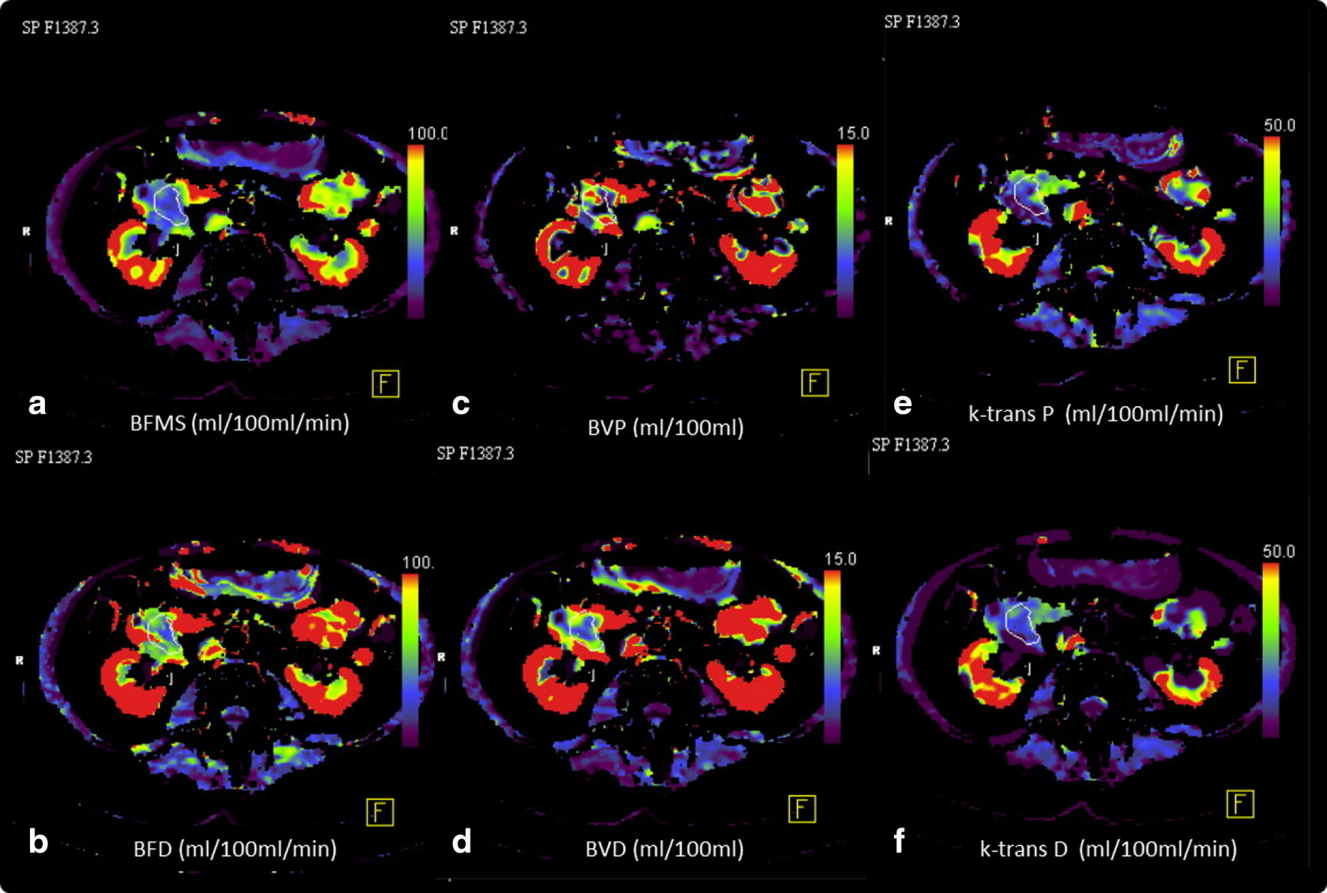
Example of a VPCT study a 62 year old patient with an adenocarcinoma in the pancreatic head. **a**–**f**) Colour maps of functional parameters: **a** Blood flow according to Max. slope Mode (BFMS; 26 ml/min/100 g), **b** BF calculated with Deconvolution method (BFD 42 ml/min/100 g). **c** Blood volume calculated with Patlak Model (BVP 6 ml/100 g), **d** Blood volume calculated with Deconvolution method (BVD 9 ml/100 g), **e** K-trans calculated with Patlak Model (k-transP 16 ml/min/100 g), **f** K-trans calculated with Deconvolution method (9 ml/min/100 g)

All data sets were transferred to a dedicated workstation (Syngo MMWP, VE 36A, Siemens Healthcare, Forchheim, Germany) and quantitative data evaluation was performed with a commercial software (Syngo Volume Perfusion CT Body). Automated motion correction and noise reduction of all datasets were applied by using an integrated motion correction algorithm with non-rigid deformable registration for anatomic alignment. A circular region of interest (ROI) was placed in the abdominal aorta, which provided the arterial input function for the computations. A second volume of interest (VOI) was placed in the pancreatic carcinoma for calculating the tumour perfusion. The VOI were chosen as large as possible and placed to avoid vessels and artefacts in a slice by slice approach (Fig. 1). For perfusion calculation we used two mathematical calculation methods (models): Compartmental analytic models (maximum slope (BFMS) + Patlak analysis (BVP, k-transP) vs. deconvolution model (BFD, BVD and k-transD). These two different kinetic calculation software programs used are both FDA approved and are part of the post-processing software recommended by the vendor. Perfusion parameters (BF, BV and k-trans) of both models were compared.

### Statistical analysis

Statistical calculation was performed using JMP 11 (SAS Institute, Cary NC, USA) and SPSS 23 (Chicago, IL, USA). Mean values of BF, BV and *k*-trans as well as of their standard deviations (SD) are reported. Differences between the functional parameters were compared using the Wilcoxon matched-pairs test and Bland-Altman plots. Second, the mean difference, SD of the differences and the 95% limits of agreement (mean difference − 2 × SD and mean difference + 2 × SD) were calculated for each of the perfusion parameters. If Bland-Altman plots showed a linear relation a linear regression analysis was performed. Statistical significance was established at a *P* value <0.05.

Agreement between the different perfusion measures was assessed using the intra-class correlation coefficient (ICC) as discussed by Shrout and Fleiss [17]. A value close to 1 indicates excellent agreement between the two readings. Additionally the Pearson’s linear correlation coefficient was calculated for the corresponding functional parameter values obtained with the different methods. Parameters for different histological grading were compared using an ANOVA analysis.

## Results and discussion

Forty eight Patients (28 men, 20 women, mean age: 69, range: 49–85 years) with untreated pancreatic adenocarcinoma were outlined and analysed. In total 28/48 tumours were localized in the head, 10/48 in the corpus and 10/48 in the pancreatic tail. In 42 patients biopsy-proven pathologic tumour grading was available (4 G1, 24 G2, 14 G3). The mean tumour size (measured the largest diameter) was 4.0 cm (range: 1.7–8.1 cm).

The calculated values of the *blood flow* (mL/100 g tissue/’) in the compartment analysis (maximum slope, BFMS) were significantly lower than the calculated values in the deconvolution (BFD) method, with values of 20.4 ± 9.7 ml/min/100 g (range: 1.6–48.22 ml/min/100 g) in the maximum slope respectively 36.9 ± 16.0 ml/min/100 g (range: 5.54–68.61 ml/min/100 g) in the deconvolution method (*p* < 0.004) (Table 1).

**Table 1:** Functional VPCT values for adenocarcinomas

Parameter	Max. slope	Patlak	Deconvolution	Mean Difference	95% limits of agreement	*p*-value^a^
BF (ml/min/100 g)	20.4 ± 9.7		36.9 ± 16.0	16.5 ± 8.9	−0.9;33,9	<0.001
BV (ml/100 g)		5.6 ± 5.5	7.3 ± 4.7	−1.7 ± 4.6	−10.7;7.3	0.004
k-trans (ml/min/100 g)		18.9 ± 9.8	12.4 ± 8.2	6.5 ± 5.8	−4.9;17.9	<0.001

Mean values of functional perfusion parameters for *n* = 48 adenocarcinomas obtained with Maximum slope-, Patlak- and deconvolution models

^a^for Wilcoxon matched-pairs test

*Abbreviations*: BF blood flow, *BV* blood volume, *G*1-3 tumor grading

The calculations of *blood volume* (BVMS vs. BVD) (mL/100 g tissue) showed similar results, with significantly higher values in the deconvolution method (*p* = 0.004). We calculated a mean blood volume value of 5.6 ± 5.5 ml/100 g (range: 0.81–25.71 ml/100 g) with the compartment analysis (Patlak analysis), whereas the deconvolution method yielded a mean value of 7.3 ± 4.7 ml/100 g (range: 1.56–21.58 ml/100 g). This difference was also significant (*p* < 0.001).

The calculated vessel wall *permeability* (*k*-*transP* vs. *k*-*transD*, mL/100 g tissue) showed a different trend. This parameter yielded higher values for the compartment analysis (Patlak analysis) than for the deconvolution method, with values of 18.9 ± 9.8 (range: 4.86–41.77), respectively 12.4 ± 8.2 (range: 0.5–30.34) (*p* < 0.001).

**Fig. 2:**
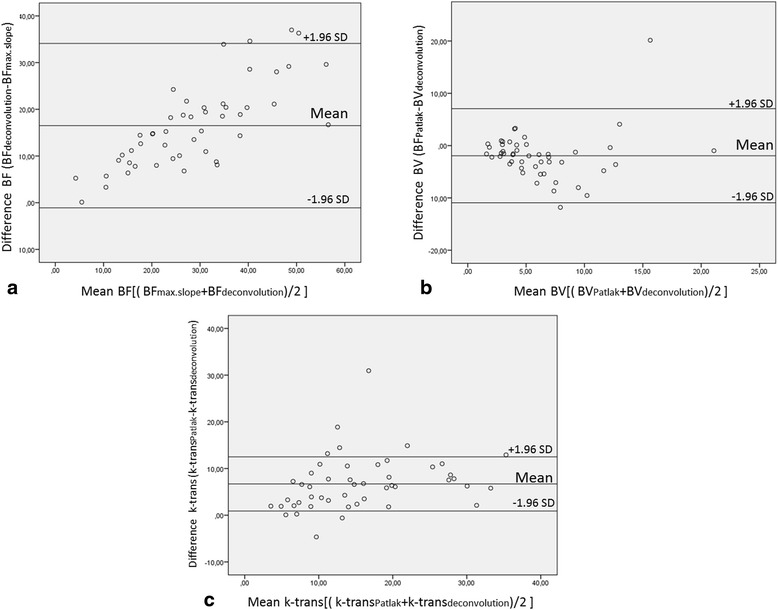
Bland-Altman plots of Blood flow (**a**, BF), Blood volume (**b**, BV) and Permeability (**c**, k-trans). **a** The plot confirms higher values for BFD compared to BFMS with higher differences for higher absolute values. **b** Lower absolute values for BVP compared with BVD. There are no systematic deviations in high or low absolute values. **c** K-transD shows lower values than k-transP without systematic deviations throughout the range of the values. *Abbreviations*: BFD = blood flow calculated with deconvolution; BFMS = blood flow calculated with maximum slope; BVD = blood volume calculated with deconvolution; BVP = blood volume calculated with Patlak; k-trans = permeability surface area product, or k-trans; k-trans; D = k-trans calculated with deconvolution; k-trans P = k-trans calculated with Patlak

**Fig. 3:**
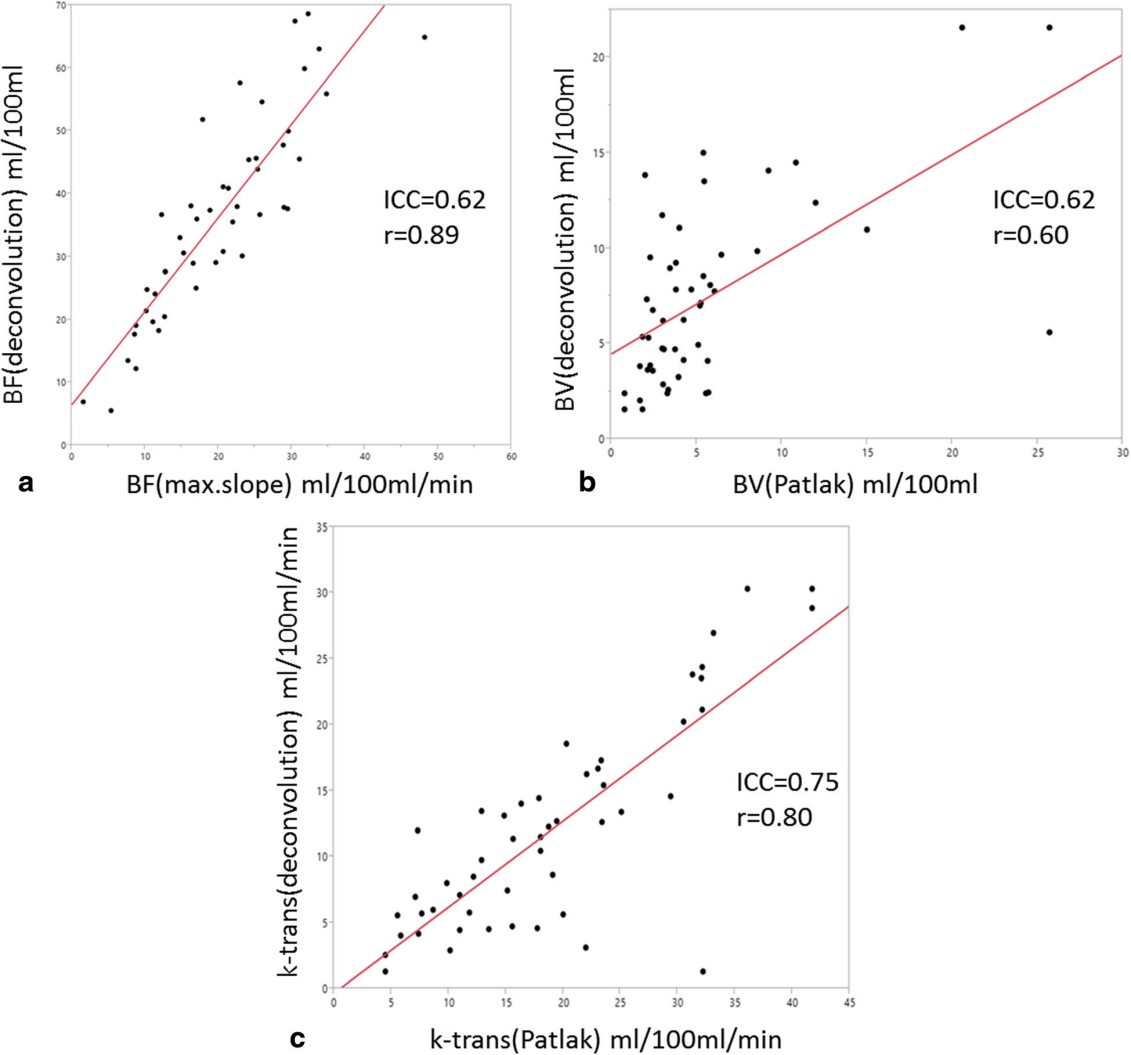
Plotted data of thee corresponding functional parameters calculated with both methods. *Red line* is the linear regression function for each functional parameter with indicated ICC and Pearsons r. **a** Linear regression analysis for BFMS vs. BFD showed a strong correlation and a good linear fit with a resulting slope of 1.5. (**b** and **c**) Despite significant differences between Patlak- and deconvolution models (BVP vs. BVD and k-transP vs. k-transP), moderate to good correlation between the functional perfusion-based parameters with regard to ICC and Pearson’s linear correlation coefficient were found. *Abbreviations*: BFD = blood flow calculated with deconvolution; BFMS = blood flow calculated with maximum slope; BVD = blood volume calculated with deconvolution; BVP = blood volume calculated with Patlak; k-trans = permeability surface area product, or k-trans; k-trans; D = k-trans calculated with deconvolution; k-transP = k-trans calculated with Patlak

The Bland-Altman plots of the perfusion parameters showed no systematic errors for higher or lower mean values in BV and k-trans (Fig. 2). For BF, Bland-Altman Plots showed a linear relation with higher differences at high absolute values. Subsequent linear regression analysis showed a good linear fit with a resulting slope of 1.5. (Fig. 3a).

Despite significant differences in the calculated perfusion parameters between the different models, moderate to good correlation between the functional perfusion-based parameters with regard to ICC and Pearson’s linear correlation coefficient (Table 2 and Fig. 3) were found.

**Table 2:** Correlation between VPCT parameters obtained with different calculation models

Parameter	ICC	Pearsons r
Blood Flow (BF) (ml/min/100 g)	0.62	0.89
Blood Volume (BV) (ml/100 g)	0.62	0.60
Permeabilty (k-trans) (ml/min/100 g)	0.75	0.80

ICC and Pearson’s linear correlation coefficient of the functional parameters obtained with Maximum slope-, Patlak- and deconvolution models

*Abbreviations*: *BF* blood flow, *BV* blood volume, *G*1-3 tumor grading

Among the three histological differentiation subgroups (G1-G3), functional parameters did not vary significantly. No functional fingerprints could be established for less differentiated lesions. This was consistent between the mathematical models (Table 3).

**Table 3:** Influence of histologic grading on perfusion parameters

	BF		BV		k-trans	
Grading	Max.slope (BFMS) (ml/min/100 g)	Deconvolution (BFD) (ml/min/100 g)	Patlak (BVP) (ml/100 g)	Deconvolution (BVD) (ml/100 g)	Patlak (k-transP) (ml/min/100 g)	Deconvolution (k-transD) (ml/min/100 g)
G1 (*n* = 4)	20.6 ± 8.6	33.5 ± 10.3	8.9 ± 11.3	6.4 ± 1.3	19.3 ± 4.5	11.5 ± 6.4
G2 (*n* = 24)	21.9 ± 10.4	37.7 ± 16.6	5.5 ± 4.5	7.9 ± 5.0	21.0 ± 10.2	13.7 ± 8.8
G3 (*n* = 14)	19.1 ± 8.4	35.6 ± 13.9	3.9 ± 2.4	6.1 ± 3.9	17.4 ± 8.7	11.9 ± 7.4
*p*-value (ANOVA)	0.71	0.94	0.42	0.68	0.55	0.71

Influence of histologic grading on perfusion parameters in *n* = 42 patients with histologic grading available

*Abbreviations*: *BF* blood flow, *BV* blood volume, *G*1-3 tumor grading

CT perfusion imaging is evolving rapidly. Based on a recently published review recommending how to optimize CT-perfusion protocols in the light of current knowledge about strengths and limitations of the available mathematical calculation models and suggesting ways to standardize the state of the art examinational protocols, the aim of the study was to make a comparison of results obtained with the recommended perfusion protocol and available calculation methods and report about the magnitude of obtained perfusion values as well as of their ranges and correlations with the histologic differentiation grade [18].

Our results clearly show that quantification of pancreatic carcinoma perfusion is feasible with the proposed VPCT-examinational protocol and that the measured perfusion values are in line with those reported in previous works dealing with this issue and using comparable perfusion protocols [19]. Moreover, they confirm the knowledge that results of the two used mathematical calculation methods significantly differ from each other and are thus not directly interchangeable, but that the magnitude of their pairs of calculated parametric values stays in constant relation to each other for BV and k-trans values. BF values show a linear relationship with higher values for the deconvolution method and a linear relationship with a slope of 1.5. These differences are systematic differences explainable by the different underlying mathematical models. The Maximum slope model underestimates BF because venous outflow is not considered by the model. This error is proportional to the absolute BF. Accordingly the relation between BFMS and BFD is a constant ratio. The presented cohort comprising 48 cases is currently the largest VPCT series of pancreatic adenocarcinoma. There is a growing amount of data from the literature reporting perfusion values of the pancreas. However this data is very heterogeneous with regard to technical parameters and included subjects. Accordingly literature values are hard to compare. In line with previous reports, in our study, blood flow values were found to be significantly lower when calculated with the compartment model compared to values obtained with the deconvolution method. The same trend was observed also for the measured absolute values of blood volume which proved to be significantly lower when calculated with the compartment model vs. the deconvolution method. Expectedly, the calculated k-trans values were lower for the deconvolution method vs. the two-compartment model (Patlak model). Xu et al. reported blood flow and blood volume values in the tumour tissue of 29.5 ml/min/100 g and 59.72 ml/100 g, respectively, both measured with the deconvolution method [20]. The blood flow value was thus lower in their study, but still in the same range with our deconvolution measured values. However, the blood volume was noticeably higher than in our study (59.7 ml/100 g vs. 7.3 ml/100 g). Similar results were reported also by Klauß et al. who reported BF values for pancreatic adenocarcinoma calculated with the Patlak analysis that were in the same range with ours; however, calculated BV values proved again significantly higher than in our population (38.9 ml/100 g vs. 5.6 ml/100 g) [21]. These discrepancies highlight the imperative of using robust examinational protocols as well as kinetic calculation models. According to our experience, a blood volume significantly higher than the corresponding blood flow is difficult to explain, in particular in a tumour with known desmoplastic stroma and lowered vascularisation. Notably, the data of Klauß et al. was obtained using a dual-energy perfusion protocol. On the contrary, Tan et al. obtained considerably higher BF values 60 ± 15.3 ml/min/100 g using lower temporal resolution during the first pass phase [22]. In particular, in the maximum slope model, temporal resolution between the start of contrast agent administration and the peak enhancement should be kept as high as technical feasible in order to avoid false high miscalculation [18]. Accordingly, the report by Li et al. using a reduced-dose examinational CT-perfusion protocol comparable to ours yielded similar results for BF, BV as well as for k-trans using the Patlak calculation model [23]. Another essential aspect with perfusion-CT is that concerning the total examination time. This is in particular essential for the calculation of k-trans. Spira et al. demonstrated significant decrease of BV values and concomitantly significant increase of k-trans-values with progressively shortened measurement time (down to 39 s) in lung carcinomas [24]. Accordingly it can be assumed that difference between k-transD and k-transP is caused partly by the limited scan time.

The use of perfusion-CT in other tumour tissues as reported by several previous studies has already demonstrated significant differences between the different mathematical models [11–13, 25]. A study from Djuric-Stojanovic et al., examining the perfusion parameters of oesophageal carcinoma, yielded a similar tendency with blood flow values showing significantly higher values for the compartmental analysis [13]. Similar to our study on pancreatic lesions, a good correlation for the blood volume between the compartment- and the deconvolution model has also been shown for malignancy in the lung (0.86), spleen (0.9) and brain (0.79) [26].

It has been suggested that perfusion-based tumour characterisation could be a non-invasive tumour grading method in pancreatic adenocarcinoma [6, 27]. This is a highly interesting topic as tumour grading is a very important prognostic factor in patients suffering from pancreatic carcinoma [3]. However despite using a similar protocol, in the present study none of the applied models based on perfusion parameters proved able to reliably discriminate between degrees of tumour differentiation. However, even in this respect, the studies are not directly comparable as the applied parameters Peak Enhancement Intensity (PEI) which indicates the peak attenuation reached by the tissue after contrast media injection and BV vs. BF, BV and k-trans and the histologic subdivisions were different. We believe that in the above mentioned study, the temporal resolution (every 5 s) may have been too low in order to accurately determine the PEI and BF [6]. However, we agree that grading does not seem to be possible if based solely on the parameter BF. The reason for the inconclusiveness of the present data remains unclear. However there are also some concerns with regard to histologic analysis which might have led to these results. At first the histologic specimen is usually very small and limited to a small part of the tumor. It is known that the pancreatic adenocarcinoma consists of several parts of varying dedifferentiation [15]. Accordingly, the histologic specimen might not be representative for the whole tumour. Second, the perfusion values in pancreatic adenocarcinoma are generally very low, sometimes only slightly above noise level. By this, the measurement of relative differences within this group is intrinsically difficult as differences are expected to be very small.

## Conclusions

In summary, both perfusion calculation methods seem to be applicable, but cannot be directly compared. Nevertheless, the magnitude of pairs of parametric values is in constant relation to each other enabling the use of any of these methods for clinical trials.

## Abbreviations

BF: Blood flow
BFD: Blood flow calculated with deconvolution
BFMS: Blood flow calculated with maximum slope
BV: Blood volume
BVD: Blood volume calculated with deconvolution
BVP: Blood volume calculated with Patlak
ICC: Intraclass correlation coefficient
k-trans: Permeability surface area product, or k-trans
k-transD: k-trans calculated with deconvolution
k-transP: k-trans calculated with Patlak
MS: Maximum-slope
NECT: Non-enhanced abdominal low-dose CT
PA: Patlak analysis
PEI: Peak enhancement
ROI: Region of interest
SD: Standard deviations
VOI: Volume of interest
VPCT: Volume perfusion CT
